# The Effect of Aerobic Exercise and Low-Impact Pilates Workout on the Adaptive Immune System

**DOI:** 10.3390/jcm11226814

**Published:** 2022-11-17

**Authors:** László Balogh, Krisztina Szabó, József Márton Pucsok, Ilona Jámbor, Ágnes Gyetvai, Marianna Mile, Lilla Barna, Peter Szodoray, Tünde Tarr, Zoltán Csiki, Gábor Papp

**Affiliations:** 1 Institute of Sport Sciences, University of Debrecen, H-4032 Debrecen, Hungary; 2 Division of Clinical Immunology, Institute of Internal Medicine, Faculty of Medicine, University of Debrecen, H-4032 Debrecen, Hungary; 3 Department of Immunology, Oslo University Hospital, Rikshospitalet, 0372 Oslo, Norway

**Keywords:** aerobic exercise, Pilates, adaptive immunity, regulatory T cell, interleukin-10

## Abstract

Growing evidence indicates the pronounced effects of physical activity on immune functions, which may largely depend on the type of exercise, intensity, and duration. However, limited information is available regarding the effects of low-impact exercises, especially on the level of adaptive immune system. Our study aimed to investigate and compare the changes in a broad spectrum of lymphocyte subtypes after 14 weeks of aerobic-type total-body-shaping workouts (TBSW) and Pilates workouts (PW) among healthy individuals. We determined the percentages of peripheral natural killer cells and different T and B lymphocyte subtypes with flow cytometry. At the end of the exercise program, significant changes in naïve and memory lymphocyte ratios were observed in TBSW group. Percentages of naïve cytotoxic T (Tc) cells elevated, frequencies of memory Tc and T-helper cell subsets decreased, and distribution of naïve and memory B cells rearranged. Proportions of activated T cells also showed significant changes. Nonetheless, percentages of anti-inflammatory interleukin (IL)-10-producing regulatory type 1 cells and immunosuppressive CD4^+^CD127^lo/−^CD25^bright^ T regulative cells decreased not only after TBSW but also after PW. Although weekly performed aerobic workouts may have a more pronounced impact on the adaptive immune system than low-impact exercises, both still affect immune regulation in healthy individuals.

## 1. Introduction

Studies of the last decades on the role of a sedentary lifestyle in developing chronic diseases have highlighted the critical importance of regular physical activity and exercise in maintaining health and reducing risk of several disorders. Lower physical activity levels correlate with the development of many chronic diseases and unhealthy conditions, such as obesity, hypertension, cardiovascular diseases, type 2 diabetes mellitus, metabolic syndrome and other chronic inflammatory disorders [1,2], while regular physical activity and exercise have beneficial effects in these conditions [3]. Growing evidence also underlines the preventive role of a physically active lifestyle in infections and other immune-mediated diseases by improving immune competency and regulation [4,5,6]. However, the immunological effects of exercise and its interpretation are still controversial in some cases. It is well known that a single bout of exercise is associated with an initial enhancement in peripheral lymphocyte numbers and effector immune functions, quickly followed by a brief period of immune depression that can last 3–72 h after the exercise bout [7]. The decrease in lymphocyte levels following the exercise has been partially explained by an increase in apoptosis [8]. It was reported that prolonged exercise decreases Th1 cell levels, but not Th2 cells [9], which selectivity could be explained by the increase of stress hormone levels (e.g., cortisol, catecholamines) and myokines in the peripheral blood [10]. Of note, cortisol suppresses the production of interleukin (IL)-12 of antigen-presenting cells (APC), which is a well-known stimulator of Th1 and NK cells [11]. These observations led to the creation of the so-called ‘open window’ hypothesis, which assumed that the immune system is transiently compromised after acute exercise. However, observations in animal models revealed that T cells are redeployed to the gut, lungs, and bone marrow following exercise [12]. This contributed to the development of a more up-to-date viewpoint in interpreting decreased immune cell frequencies after exercise, which might reflect the even heightened immune-surveillance and immuno-regulatory activities instead of the suppression of the immune system [13,14].

Regarding the long-lasting effects of excessive high-intensity and high-volume physical activity typically practiced by highly competitive athletes, a reduction in proportions of immunocompetent cells with effector functions and decrease of several cytokines, including IL-6, tumor necrosis factor (TNF)-α, interferon (IFN)-γ, IL-1β, IL-2, IL-8 and IL-10, were reported [15,16]. Save for the frequent and arduous bouts of exercise that far exceed recommended physical activity guidelines, there is no doubt that an active lifestyle with regular exercise results in improved immune functions and reduction of systemic inflammatory activity. Former observations reported increased IL-2 production, T-cell proliferation, NK cell cytotoxic activity and enhanced vaccine responses [17,18]. Furthermore, it has also been suggested that repeated bouts of exercise may delay immunosenescence by limiting the accumulation of memory T cell subsets and increasing the frequency of circulating naive T cells [19].

Low-impact workouts, such as Pilates, are beneficial to individuals who cannot perform intensive or moderate-intensity exercises due to chronic health conditions. The purpose of the movements is to increase core strength and muscle balance, improve flexibility and posture; meanwhile, practicing effective breathing exercises during Pilates can encourage relaxation and reduce stress [20]. Although we have a rapidly expanding knowledge on the immunological effects of high- and moderate-intensity workouts, there is still only limited information available about the effects of low-impact exercises, especially on the level of the adaptive immune system. Therefore, our study aimed to examine and compare the changes in a broad spectrum of lymphocyte subtypes after aerobic-type total-body shaping and low-impact Pilates workouts among healthy individuals.

## 2. Materials and Methods

### 2.1. Participants

Thirty-two healthy female university students were enrolled in the present study, who participated in general physical education (PE) classes arranged by the Institute of Sport Sciences, University of Debrecen. Each volunteer completed an assessment of dietary and exercise habits questionnaire before and after the exercise program. Participants enrolled in the study were non-smokers. They were instructed to refrain from any physical activity, special diet, and vitamin supplements for at least three months before the investigation. Moreover, exclusion criteria included ongoing viral or bacterial infection, allergic or autoimmune disease, chronic disease treated with continuous drug therapy, cancer, alcohol or drug addiction, pregnancy or breastfeeding, psychiatric illness, insufficient cooperation skills and dietary changes or usage of dietary supplements during the entire study.

Informed written consent was obtained from all subjects enrolled in the investigation. The study was conducted according to the guidelines of the Declaration of Helsinki and approved by the Ethics Committee of the University of Debrecen (protocol number: 4839-2017, date of approval: 26 June 2017) and the Policy Administration Services of Public Health of the Government Office (registration number: 25040-4/2017/EÜIG, date of approval: 4 September 2017).

### 2.2. Exercise Protocols

Fourteen students (median age; min–max: 21 years; 18–25) voluntarily participated in a Pilates exercise routine, while eighteen students (median age; min–max: 21 years; 20–29) voluntarily participated in an aerobic-type total-body-shaping workout routine. Participants started their assigned activities after completing primary laboratory data collection. Every session was supervised by a physical education professional specialized in the relevant exercise. Each activity included a 90 min long session per week, for 14 weeks in total. Pilates workouts included floor-based exercises on a mat, focusing on controlled, low-intensity movements, stretching, and breathing. Each session consisted of 20 min of warm-up, stretching, 50 min of musculoskeletal exercises, and 20 min of stretching and cool-down exercises. Each session included ten basic exercises, as described by Joseph Pilates [21], including the pelvic curl, the chest lift, the chest lift with rotation, the spine twist supine, single leg stretch, the roll-up, the roll-like-a-ball, leg circles, all fours, and the back extension. Total body stretching exercises included quad stretch, standing hamstring stretch, chest and shoulder stretch, upper back stretch, biceps stretch, shoulder stretch, seated side stretch and triceps stretch.

The aerobic-type total-body-shaping workout routine included three sections: 20 min of warm-up, 50 min of aerobic-type musculoskeletal exercises, and 20 min of stretching and cool-down exercises. The warm-up session incorporated squats, high knees, leg swings, lunges, plank walk-outs, arm circles, standing toe taps, jumping jacks, butt kicks and hip circles exercises. The aerobic exercise routine consisted of the following exercises: 5 min of step-tap, 7 min of tapping, 6 min of side-steps, 6 min of grapevine with side-steps, 7 min of arm-pumps, 6 min of forward-backward walk, 6 min of heel-steps and 7 min of sit-ups. Sessions of week one to four incorporated exercises with light-to-moderate intensity [50 to 70% of maximum heart rate (HRmax)]. From week five, a higher intensity level (75–85% of HRmax) was applied; exercise intensities were monitored via Polar Team Pro System (Polar Electro, Kempele, Finland). Finally, a cool-down and stretching session was incorporated.

### 2.3. Blood Sampling

We collected blood samples from a peripheral vein of the upper extremity before the first workout and three days after completing the entire 14-week exercise routine at the outpatient clinic of the Division of Clinical Immunology, Institute of Internal Medicine, Faculty of Medicine, University of Debrecen.

### 2.4. Assessment of Lymphocyte Subpopulations by Flow Cytometry

The distribution of a broad spectrum of lymphocyte subpopulations was determined by flow cytometry. Heparinized peripheral blood samples were obtained from the participants, and the whole blood was used for the experiment. Cells were stained with a combination of different fluorophore-conjugated monoclonal antibodies for 30 min at room temperature. Erythrocytes were haemolysed with a 0.2% solution of formic acid. The cells were then washed twice, fixed with 1% solution of paraformaldehyde, and stored at 4°C until further assessment. Different lymphocyte subsets were analysed and identified by flow cytometry using the antibody panel for cell surface staining described in Table 1. Isotype controls (IgG1 antibody cocktail, from Beckman Coulter Inc., Brea, CA, USA) were used in all procedures. Measurements and data analysis were performed on a Coulter FC500 flow cytometer equipped with Kaluza 1.2a software (both from Beckman Coulter).

### 2.5. Determination of T Helper and T Cytotoxic Cells by Immunofluorescent Staining of Intracellular Cytokines

The distribution of Th cell subsets and Tc cells was assessed by flow cytometry. For stimulating cytokine-producing T cells, whole blood was diluted to 1:1 with saline solution and stimulated with phorbol-12-myristate 13-acetate (PMA) (25 ng/mL), ionomycin (1 μg/mL) for five hours at 37 °C in 5% CO_2_ milieu. Golgi Stop brefeldin A (10 μg/mL) (all from Sigma Aldrich, St. Louis, MO, USA) was added to the culture for the last 4 h. After cell surface staining, cells were fixed and permeabilized with Intraprep^TM^ permeabilization reagent (Beckman Coulter) according to the manufacturer’s instructions. Then, intracellular cytokine staining was carried out with a combination of fluorophore-conjugated monoclonal antibodies. Cells were evaluated on a Coulter FC500 flow cytometer and data were analysed with Kaluza 1.2a software (both from Beckman Coulter). Isotype-matched antibodies were used in all experiments. All antibodies used for this measurement and the determined T cell subpopulations are summarized in Table 2.

### 2.6. Statistical Analysis

Data were statistically analysed with GraphPad Prism 8 software (Graphpad Software, San Diego, CA, USA). Kolmogorov–Smirnov and Shapiro–Wilk normality tests were used to determine the distribution of data. In the case of Gaussian distribution, a two-tail paired t-test was used; otherwise, if the data set differed from the normal distribution, the Wilcoxon test was performed. Repeated measures two-way ANOVA with Bonferroni correction for multiple comparisons was used to assess data in lymphocyte panels analysing different cell subsets. Differences were considered statistically significant at *p* < 0.05.

The sample size was calculated based on data from previous studies with approximate changes in the percentages of different lymphocyte subpopulations during physical activity [22,23]. In order to determine a medium effect size (effect size of Cohen’s d = 0.65), we estimated we would need to enroll at least 21 individuals in each groups to obtain 80% power (1-β) and 5% significance level (α = 0.05, two-tailed) in a t-test with matched pairs. Sample size calculation was performed using G*Power v3.1.9.7. Software (University of Düsseldorf, Düsseldorf, Germany). Unfortunately, several students did not attend for post-test after the 14-week exercise program and were lost to follow-up (PW, n = 6; TBSW, n = 1).

## 3. Results

### 3.1. Data Describing Study Population

The median age of the participants was 21 years (min–max: 18–29 years) at the time of the study. Based on body weight and height data, all participants demonstrated a normal Body Mass Index (22.31 ± 1.75) at enrolment, which remained essentially unchanged until the end of the study (22.43 ± 1.82).

### 3.2. Quantification of Different Lymphocyte Subpopulations before and after the Exercise Routine

**Figure 1:**
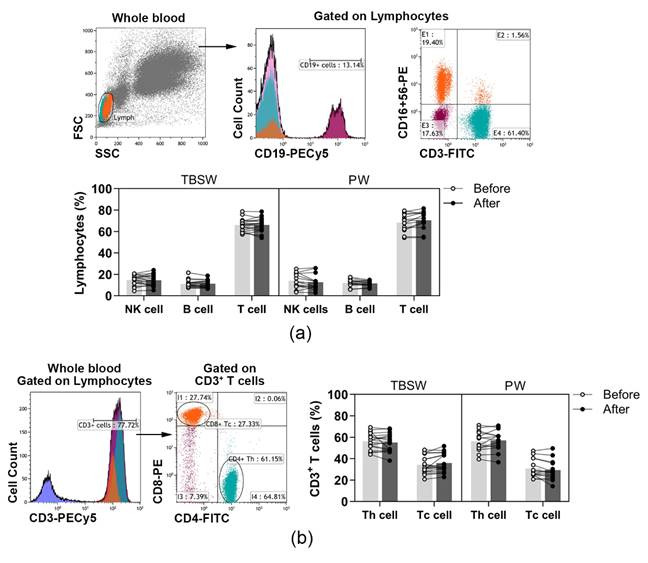
The distribution of peripheral lymphocyte subsets in young women before and after the exercise course. The whole blood of 32 healthy volunteers was stained with labelled monoclonal antibodies, as described previously. According to the exercise courses, the trainees were divided into total-body-shaping workout (TBSW, n = 18) and Pilates workout (PW, n = 14) subgroups. (**a**) Representative dot plots and a histogram show the gating strategy of CD3^−^CD16^+^CD56^+^ NK, CD19^+^ B, and CD3^+^CD16^−^CD56^−^ T cell populations. The bar chart shows the percentages of NK, B, and T cells. (**b**) The representative histogram shows the gating of CD3^+^ cells, and the dot plots demonstrate the distribution of CD4^+^ Th and CD8^+^ Tc cells. The bar chart indicates the frequencies of Th and Tc cells. Data analysis was performed with repeated measures two-way ANOVA followed by Bonferroni multiple comparisons test. Each data point represents an individual subject, while bars show the mean values.

A wide spectrum of peripheral immune-competent cells was analysed with a flow cytometer in healthy volunteers’ blood as summarized in Table 1. According to the variety of exercise courses, we divided the subjects into two subgroups, total body shaping and Pilates. Basic lymphocyte subpopulations, were identified according to their cell surface markers. T, B and NK cells were quantified as to their percentages in lymphocytes, while Tc and Th cells were assessed as to their ratio in CD3^+^ T cells. According to our results, the distribution of basic lymphocyte subsets did not change significantly during both the total-body-shaping and Pilates workout programs (Figure 1a,b).

**Figure 2:**
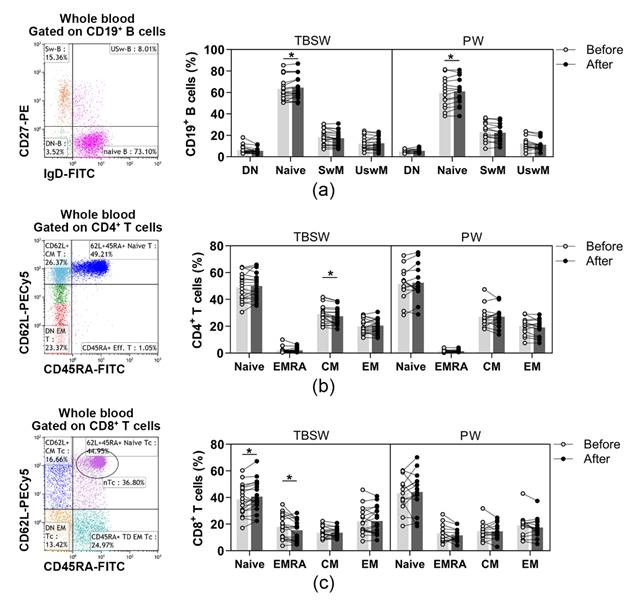
Assessment of naïve and memory lymphocyte subsets in young women after a 14-week workout program. The whole blood of 32 healthy individuals was stained with fluorochrome-labeled monoclonal antibodies, as described previously. According to the exercise courses, the trainees were divided into total-body-shaping workout (TBSW, n = 18) and Pilates workout (PW, n = 14) subgroups. (**a**) Representative dot plot indicates the distribution of IgD^+^CD27^−^ naïve, IgD^−^CD27^−^ double negative (DN), IgD^−^CD27^+^ switched, and IgD^+^CD27^+^ un-switched memory B cells. The bar chart shows the percentages of B cell subsets. (**b**) Representative dot plot demonstrates the distribution of CD45RA^+^CD62L^+^ naïve, CD45RA^−^CD62L^−^ effector memory (EM), CD45RA^−^CD62L^+^ central (CM) and CD45RA^+^CD62L^−^ effector memory (EMRA) Th cells. The bar chart indicates the frequencies of Th cell subpopulations. (**c**) Representative dot plot demonstrates the distribution of CD45RA^+^CD62L^+^ naïve, CD45RA^−^CD62L^−^ EM, CD45RA^−^CD62L^+^ CM and CD45RA^+^CD62L^−^ EMRA Tc cells. The bar chart indicates the frequencies of Tc cell subpopulations. Repeated measures two-way ANOVA with Bonferroni correction for multiple comparisons was used. Each data point represents an individual subject, while bars show the mean values. Statistically significant differences are indicated by * *p* < 0.05.

In addition, naïve and memory lymphocyte subpopulations were distinguished within B, Th and Tc cells. B cell subsets were quantified as to their percentages in CD19^+^ lymphocytes, and Th subpopulations were determined as their ratio in the CD4^+^ cells. In contrast, Tc cell subsets were quantified as to their frequencies in CD8^+^ cells. Regarding naïve and memory B cell subsets, only the percentages of naïve B cells were significantly elevated both in the total-body-shaping (63.179 ± 11.048 vs. 64.537 ± 11.173; *p* = 0.0297) and in the Pilates subgroup (59.656 ± 13.871 vs. 60.996 ± 13.008; *p* = 0.0417) by the end of the exercise course (Figure 2a). In the case of naïve and memory Th cells, we observed that the proportions of central memory (CM) Th cells differed significantly (29.151 ± 7.010 vs. 27.468 ± 6.716; *p* = 0.0363) exclusively in the total-body-shaping subgroup, while the other cell subsets within the group, as well as in Pilates, did not change significantly (Figure 2b). There was no significant difference in naïve and memory Tc cell subsets in Pilates group. A statistically significant increase was found in the percentages of naïve Tc (38.544 ± 11.453 vs. 40.777 ± 11.949; *p* = 0.0428), and a significant decrease was detected in the ratio of CD45RA^+^ effector memory (EMRA) Tc (17.779 ± 9.124 vs. 15.418 ± 7.892; *p* = 0.0284) cells in the total-body-shaping workout group (Figure 2c).

### 3.3. Determination of Cells with a Regulatory Function in the Innate and Adaptive Immune Response after a 14-Week Exercise Routine

**Figure 3:**
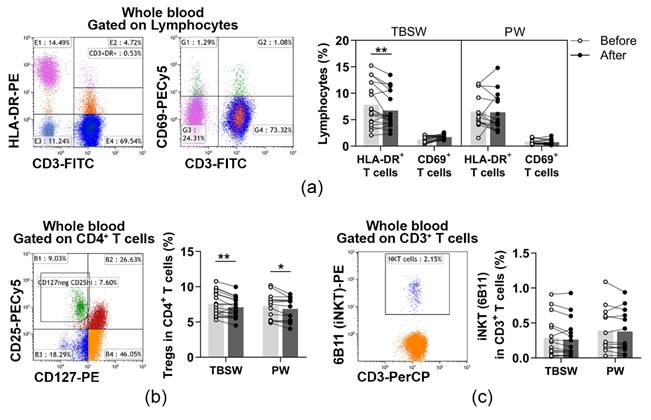
Measurement of lymphocytes with a regulatory function in young women after a 14-week exercise program. The whole blood of 32 healthy volunteers was stained with labelled monoclonal antibodies, as described previously. According to the exercise courses, the trainees were divided into total-body-shaping workout (TBSW, n = 18) and Pilates workout (PW, n = 14) subgroups. (**a**) Representative dot plots demonstrate the identification of CD3^+^HLA-DR^+^ late-activated and CD3^+^CD69^+^ early activated T cells. The bar chart indicates the frequencies of activated T cells. (**b**) Representative dot plot indicates the identification of CD4^+^CD25^bright^CD127^lo/−^ Treg cells within CD4^+^ T cells. The bar chart indicates the ratio of Treg cells. (**c**) Representative dot plot shows the determination, and the bar chart indicates the frequencies of CD3^+^6B11^+^ NKT cells. 6B11 is referred to as the invariant NKT (iNKT) marker. Repeated measures two-way ANOVA with Bonferroni correction for multiple comparisons was used for activated T cells, and paired T-test or Wilcoxon test was used for the statistical analysis of NKT and Treg cells. Each data point represents an individual subject, while bars show the mean values. Statistically significant differences are indicated by * *p* < 0.05; ** *p* < 0.01.

In the peripheral blood of young women, CD69^+^ early activated T cells and HLA-DR^+^ late-activated T cells were determined. Their distribution was quantified as to their percentages in the lymphocyte population. When we analysed the ratio of activated T cells, significant differences were only detected in the total-body-shaping subgroup. The percentages of late-activated T cells were significantly decreased (7.804 ± 4.110 vs. 6.750 ± 3.392; *p* = 0.0031) compared to baseline values (Figure 3a).

Additionally, CD4^+^CD127^lo/−^CD25^bright^ Treg cells and CD3^+^6B11^+^ NKT cells were also identified before and after the exercise program. Their division was assessed as to their frequencies in CD4^+^ cells, and in CD3^+^ cells, respectively. When we analysed the distribution of Treg cells, we found that it was significantly decreased in the total-body-shaping (7.554 ± 1.723 vs. 7.082 ± 1.415; *p* = 0.0032) as well as in the Pilates (7.266 ± 1.771 vs. 8.861 ± 1.642; *p* = 0.0142) subgroups (Figure 3b). The ratio of NKT cells did not change significantly by the end of the exercise course (Figure 3c).

### 3.4. Assessment of Peripheral T Helper Subsets and Cytotoxic T Cells before and after the 14-Week Training

**Figure 4:**
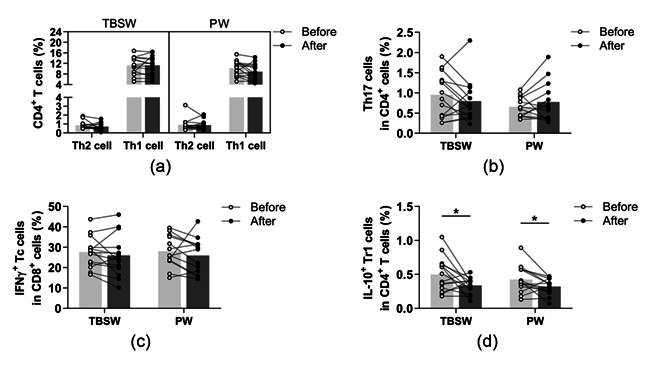
Determination of T helper and T cytotoxic cells with intracellular cytokine analysis in young women before and after the exercise program. The whole blood of 32 healthy participants was stimulated for 5h and stained with monoclonal antibodies with the intracellular staining method described previously. According to the exercise courses, the trainees were divided into total-body-shaping workout (TBSW, n = 14) and Pilates workout (PW, n = 14) subgroups. (**a**) Frequencies of IFN-γ^+^ Th1 and IL-4^+^ Th2 cells. (**b**) Percentages of IL-17^+^ Th17 cells. (**c**) The ratio of IFN-γ-producing Tc cells. (**d**) Proportions of IL-10-producing type-1 regulatory (Tr1) cells. A paired T-test was used. Each data point represents an individual subject, while bars show the mean values. Statistically significant differences are indicated by * *p* < 0.05.

The identified phenotypes of different CD4^+^ T cell subpopulations and CD8^+^ cytotoxic T cells are summarized in Table 2. All cell subsets were quantified as to their percentage in the CD4^+^ or CD8^+^ lymphocyte population. We found no significant differences in the ratio of peripheral blood Th1, Th2, Th17, and Tc cells in both exercise groups (Figure 4a–c). However, the ratio of Tr1 cells was significantly diminished in the total-body-shaping (0.499 ± 0.256 vs. 0.335 ± 0.115; *p* = 0.0420) as well as in the Pilates groups (0.426 ± 0.200 vs. 0.319 ± 0.120; *p* = 0.0362) compared to baseline values (Figure 4d).

## 4. Discussion

A properly functioning immune system is essential for the host’s continuing survival by maintaining a well-balanced defence against foreign organisms and protection from endogenous altered or virally transformed cells. However, besides the genetic and endogenous background, numerous lifestyle and environmental factors fundamentally affect immune functions. Among these factors, physical activity becomes the focus of scientific interest due to its multifaceted effects on immunity. Previous studies concerned with the effects of exercise on the immune system have focused mainly on the impact of acute bouts of exercise and the chronic influences of workout programs, especially in athletes. On the contrary, the immunological effects of low-impact exercise, such as Pilates, are definitely not in the focus of immunological research. Therefore, only limited results are available regarding the impacts of Pilates on innate immunity [22], and to our best knowledge, changes in the elements of the adaptive immune system have not been previously investigated.

Therefore, in our present study, we focused on the effects of Pilates workouts and aerobic-type total-body-shaping exercises performed for 90 min once a week on a broad spectrum of immune competent cells of the adaptive immune system. Importantly, acute changes induced by intense physical exercise may last at least 24 h, and even moderate acute exercise induces significant immune alterations for several hours [10]. Therefore, we carried out the laboratory measurements 3 days after the last workout to exclude the distorting effects of early immunological changes (e.g., postexercise lymphocytopenia).

Our knowledge is still limited regarding the long-term effects of physical exercise on B cells. Previously, studies on brief or prolonged exercise reported that the number of total B cells follows the aforementioned changes of the total lymphocyte population. After a short increase during and immediately after exercise, it falls below pre-exercise levels and then returns to basal level within 24 h [24]. Regarding long-term changes in B-cell-mediated humoral immune responses, previous studies demonstrated an elevated salivary IgA secretion after a 3-month Pilates exercise program or a 6-month-long active daily walking exercise training in elderly participants [25,26]. After regularly performed aerobic training in the elderly, increased plasma levels of IgA, IgG and IgM were reported [27], indicating the promotion of humoral immunity. Our study revealed that total-body-shaping and Pilates workouts increased the proportions of naïve B cells in young individuals. An increase in naïve B cell proportion demonstrates a beneficial rearrangement, even a rejuvenation of the available B cell population, since naïve B cells can respond to novel antigens, while on the contrary, memory B cells exhibit more restricted B cell antigen receptors [28]. Moreover, it was suggested that the ratios of immature cells and naïve B cells, which have yet to encounter antigens, were increased in peripheral blood because of their migration to the secondary lymphoid organs where antigenic screening occurs [29]. On the other hand, memory B cells either circulate in the peripheral blood or home to niches outside of the circulation, including the bone marrow, the spleen and tonsils, or several of them could be present as tissue-based memory B cells. Tracking of memory subsets revealed that switched-memory B cells mainly resided in the spleen and tonsils instead of peripheral blood at a steady state [30]. It could be assumed that the maintenance of this steady state may be further enhanced as a result of moderate training, and increased naïve B cell ratio ensures replenishment.

In the case of naïve and memory T cell distribution, we observed rearrangements similar to those we determined in B subtypes. We revealed a significant increase in the percentages of naïve Tc. Simultaneously, the ratios of effector memory Tc and central memory Th cells showed a significant decrease in the total-body-shaping group at the end of the exercise intervention study period. Although the exact mechanisms behind the changes in the distribution of naïve and memory T lymphocytes have not been elucidated yet, a possible theory may answer some questions. It is assumed that the homeostatic number of peripheral T cell repertoire is tightly regulated by a feedback mechanism. Exercise may decrease the accumulation of memory cells through their mobilization into the circulation and subsequent extravasation to peripheral tissues (e.g., mucosal surfaces of the lungs and gut or spleen and bone marrow) where they are exposed to H_2_O_2_ induced apoptosis, or where they probably encounter pathogens and carry out effector functions. Therefore, in order to maintain the proper amount of the T cell pool, a feedback mechanism increases thymic output and the accumulation of antigen-inexperienced naïve T cells at the periphery [13,31]. As the rate of thymic atrophy and loss of thymic output accelerates after puberty, the decrease in the naïve T cell pool, which is more pronounced in the CD8^+^ T cell population, compromises the recognition and combat against new pathogens [32]. The exercise-induced mobilization of naïve T cells into the circulation could ensure the maintenance of the immune response to novel pathogens [13]. Consequently, regular physical activity and exercise may be a reasonable way to delay the aging-associated alterations of the immune system and potentially increase responses to vaccinations, even in younger individuals after puberty [33]. However, in contrast to the aerobic exercise routine, we observed no changes in T cell subpopulations after the Pilates workout program. These changes suggest that aerobic-type workout sessions may be an effective intervention for the rearrangement of these cell proportions in contrast to Pilates.

When we analysed the ratio of activated T cells, significant differences were only detected in the total-body-shaping subgroup, too. The percentages of late-activated HLA-DR^+^CD3^+^ T cells were significantly decreased. However, recent studies have suggested that HLA-DR^+^CD8^+^ T cells represent natural regulatory CD8^+^ T cells, and most frequently express a high level of CD28 and low level of CD45RA [34]; therefore, to get a better view of the changes in late-activated T cell proportions, further studies are needed with a special emphasis on the changes in natural regulatory HLA-DR^+^CD8^+^ T cells, as well. We also evaluated the changes in the regulative arm of the adaptive immune system. A network of regulatory T (Treg) cells is primarily responsible for limiting immune reactions by suppressing immune activation and effector functions. There are two main types of Tregs: namely, natural CD4^+^CD25^bright^FoxP3^+^ Treg cells and induced Treg cells (iTreg), such as interleukin (IL)-10-producing T regulatory type 1 (Tr1) cells [35]. Based on previous studies, acute high-intensity exercise may significantly increase Treg cell number [23], while regular workouts of moderate intensity may lead to decreased Treg proportions in the elderly [31]. In the present study, we revealed that both aerobic-type total-body-shaping exercises and low-impact Pilates workouts decrease CD4^+^CD127^lo/−^CD25^bright^ Treg cell ratios. Additionally, we found a similar reduction in the proportions of immunosuppressive IL-10-producing Tr1 cells after both workout series. The lower ratio of Treg cells could be explained by the reference to a study with murine asthma model. Their results showed that aerobic training increased Foxp3^+^ Treg distribution in mediastinal lymph nodes and lungs; moreover, a heightened suppression capacity of Treg cells was observed compared to the sedentary control group. These results indicate that regular exercise may force the redistribution of Treg cells from the blood to the site of possible antigen exposure [36]. These novel observations shed light on the important effects of weekly performed physical exercises, including Pilates workouts, on immune regulation. Our present findings on the effects on regulatory T cell proportions might reveal an important consequence of regular physical activity in healthy individuals.

## 5. Conclusions

Our findings suggest that aerobic exercise-induced changes in the distribution of specific naïve and memory B and T cell subsets, as well as in the proportions of activated T and regulatory T subsets, may indicate a retuned immune regulation and a presumably enhanced responsiveness of the immune system. On the other hand, as a low-impact workout, Pilates may influence the proportions of regulative T cells only. Nevertheless, based on the significant effects on immune regulation, Pilates exercise may also be beneficial in maintaining appropriate adaptive immune functions. However, taking into consideration that numerous complex factors, such as hormonal status, environmental factors, etc., may affect the immune system and might influence the effects of exercise, we have to mention the lack of non-exercise control group as one of the limitations of the study. Although our results showed significant changes in B and T cell subpopulations even with a relative small sample size, more controlled investigations are needed for the deeper understanding of exercise-induced changes in the distribution of naïve and memory lymphocytes, as well as in the regulatory functions that may have an important role in preventing infections and optimizing vaccination.

## Figures and Tables

**Table 1:** List of fluorophore-conjugated monoclonal antibodies used in flow cytometry.

Antibodies	Clone	Fluorophore	Company	Lymphocyte Subsets
CD19	J3-119	PE-Cy5	Beckmann Coulter ^a^	CD3^+^ T cells,
CD3/	SK7/	FITC	BD Biosciences ^b^	CD3^−^CD16^+^CD56^+^ NK cells,
CD16+CD56	B73.1+MY31	PE		CD19^+^ B cells
CD3/ CD4/ CD8	UCHT1/ MT310/ DK25	RPE-Cy5 FITC RPE	Bio-Rad ^c^	CD3^+^CD4^+^ Th cells, CD3^+^CD8^+^ Tc cells
IgD CD27 CD19	IA6-2 1A4CD27 J3-119	FITC PE PE-Cy5	Beckman Coulter	CD19^+^IgD^+^CD27^−^ naïve, CD19^+^IgD^+^CD27^+^ UswM, CD19^+^IgD^−^CD27^+^ SwM, CD19^+^IgD^−^CD27^−^ DN B cells
CD62L	DREG56	PE-Cy5	Beckman Coulter	CD45RA^+^CD62L^+^ naïve,
CD45RA/	ALB11/	FITC		CD45RA^−^CD62L^+^ CM,
CD4	13B8.2	PE		CD45RA^−^CD62L^−^ EM,
CD45RA/	F8-11-13/	FITC	Bio-Rad	CD45RA^+^CD62L^−^ EMRA,
CD8	LT8	RPE		CD4^+^ Th and CD8^+^ Tc cells
CD3/ HLA-DR CD69	UCHT1/ WR18 FN50	FITC RPE PE-Cy5	BD Biosciences	CD3^+^CD69^+^ T cells, CD3^+^HLA-DR^+^ T cells
CD3	SK7	PerCP	BD Biosciences	CD3^+^6B11^+^ NKT cells
Inkt ^d^	6B11	PE		
CD4	RPA-T4	FITC	BD Biosciences	CD4^+^CD127^lo/−^CD25^bright^ Treg cells
CD25	B1.49.9	PE-Cy5	Beckman Coulter	
CD127	R34.34	PE		

FITC, fluorescein isothiocyanate; PE, phycoerythrin; PE-Cy5, phycoerythrin-cyanine dye 5; RPE, R-phycoerythrin; RPE-Cy5, R-phycoerythrin-cyanine dye 5; HLA, human leukocyte antigen; NK, natural killer; UswM, un-switched memory; SwM, switched memory; DN, double-negative; CM, central memory; EM, effector memory; EMRA, CD45RA^+^ effector memory; ^a^ Beckmann Coulter Inc., Brea, CA, USA; ^b^ BD Biosciences, San Diego, CA, USA; ^c^ Bio-Rad Laboratories, Hercules, CA, USA; ^d^ 6B11 monoclonal antibody reacts with invariant T-cell receptor (TCR) α-chain Vα24Jα18 expressed by natural killer T (NKT) cells and addressed as invariant NKT (iNKT) marker.

**Table 2:** The combination of monoclonal antibodies used for flow cytometry analysis of Th and Tc cell subsets.

Antibodies	Clone	Fluorophore	Company	T Cell Subsets
CD4	13B8.2	PE-Cy5	Beckmann Coulter ^a^	
IL-10	JES3-19F1	PE	BD Biosciences ^b^	IFN-γ^+^IL-4^−^ Th1 cells,
IFN-γ/	25723.11/	FITC		IFN-γ-IL-4^+^ Th2 cells,
IL-4 ^d^	3010.211	PE		IL-10^+^ Tr1 cells,
IL-17	41802	PE	R&D Systems ^c^	IFN-γ-IL17^+^ Th17 cells
CD8	4S.B3	FITC	BD Biosciences	
CD8	B9.11	PE-Cy5	Beckmann Coulter	IFN-γ^+^IL-4^−^ Tc cells
IFN-γ/	25723.11/	FITC	BD Biosciences	
IL-4	3010.211	PE		

FITC, fluorescein isothiocyanate; PE, phycoerythrin; PE-Cy5, phycoerythrin-cyanine dye 5; IFN, interferon; IL, interleukin, Th, T helper; Tc, cytotoxic T; Tr1, regulatory type-1; ^a^ Beckmann Coulter Inc., Brea, CA, USA; ^b^ BD Biosciences, San Diego, CA, USA; ^c^ R&D Systems, Minneapolis, MN, USA; ^d^ Anti-human IFN-γ FITC/IL-4 PE two-color direct immunofluorescence reagent.

## Data Availability

The data presented in this study are available in the article’s Figures and Tables.
